# Genomic Characterization of Bacterial Isolates from Aerobic Fermentation of Oolong Tea Dregs: Antimicrobial Resistance, Virulence, and Lignocellulose-Degrading Potential

**DOI:** 10.3390/foods15183201

**Published:** 2026-09-10

**Authors:** Ziheng Zeng, Ren Ke, Marsena Jasiel Ismaiah, Guanming Ye, Xiaoqing Yu, Shuning Lan, Jiarui Wang, Han Yu, Huiyu Liu, Kin Sum Leung, Lu Zhang, Jetty Chung-Yung Lee, Olivier Habimana

**Affiliations:** 1 School of Biological Sciences, The University of Hong Kong, Pokfulam, Hong Kong; u3637449@connect.hku.hk (Z.Z.); u3637450@connect.hku.hk (R.K.); imjs12@connect.hku.hk (M.J.I.); u3637252@connect.hku.hk (G.Y.); u3637293@connect.hku.hk (X.Y.); u3644013@connect.hku.hk (S.L.); u3643884@connect.hku.hk (J.W.); lks612@hku.hk (K.S.L.); lzhang17@hku.hk (L.Z.); jettylee@hku.hk (J.C.-Y.L.); 2 Biotechnology and Food Engineering Program, Guangdong Technion-Israel Institute of Technology, Shantou 515063, China; han.yu@gtiit.edu.cn (H.Y.); liu09756@gtiit.edu.cn (H.L.)

**Keywords:** tea waste, comparative genomics, lignocellulose degradation, carbohydrate-active enzymes, composting

## Abstract

The global tea industry generates millions of tons of waste annually, yet the bacteria capable of degrading this recalcitrant biomass remain poorly characterized. This study performed whole-genome sequencing and comparative genomic analysis of six bacterial strains isolated from oolong tea residue fermentation: *Bacillus* sp., *Paenibacillus* sp., *Pantoea* sp., two *Pseudomonas* isolates, and *Stenotrophomonas* sp. Genome sizes ranged from 3.61 to 6.33 Mbp, with GC content from 43.2% to 66.5%. Average nucleotide identity (ANI) analysis confirmed species-level identification for *Paenibacillus* (ANI > 95%), *Pantoea* (ANI > 95%), and both *Pseudomonas* isolates (ANI > 95%), while the *Bacillus* and *Stenotrophomonas* isolates showed ANI < 95% to all reference genomes, suggesting potential taxonomic novelty requiring further characterization. Antimicrobial resistance genes were detected only in *Bacillus* (*cat86*, *dfrG*) and *Pantoea* (*oqxB*). Genome-wide MGE profiling identified 349 MGE-associated genes across all six strains, including 101 IS elements, 61 integron-associated genes, and 183 plasmid-associated genes; tetracycline resistance-associated gene homologs were additionally detected in four strains below CARD thresholds. Virulence-associated gene profiling revealed that the 32 factors identified in *Pseudomonas* isolates primarily encode motility, biofilm formation, and secretion system components, traits associated with environmental adaptation rather than pathogenicity. Carbohydrate-active enzyme (CAZyme) annotation identified conserved plant biomass-degrading capabilities across all isolates. Descriptive comparisons suggested potential differences in enzyme profiles relative to isolation time: early-stage isolates appeared to harbor more hemicellulose-degrading enzymes, while late-stage isolates showed more lignin- and polyphenol-related enzyme annotations; however, these patterns require experimental validation. This genomic resource provides a foundation for understanding bacterial adaptation to tea waste environments and informs candidate strain selection for future inoculant development studies.

## 1. Introduction

In 2023, worldwide tea production exceeded 6.7 million metric tons, with preliminary estimates for 2024 approaching 6.9 million tons, generating substantial waste from discarded tea leaves, processing byproducts, and residual agricultural biomass, with no comprehensive recovery achieved through recycling into biofertilizer or other alternative uses [1,2]. Tea waste contains abundant lignocellulosic biomass, polyphenolic substances, and leftover nutrients; yet, its resistant structure hinders effective microbial decomposition and utilization. The chemical makeup of tea waste generally consists of around 25% cellulose, 18% hemicellulose, 22% lignin, and 10–15% polyphenolic substances such as catechins, theaflavins, and tannins, which fluctuate according to the type of tea and its processing technique. This elevated lignin and polyphenol concentration necessitates enzymatic mechanisms proficient in penetrating cellulose and hemicellulose ensconced within resistant lignin-polyphenol structures [2,3]. Aerobic composting and fermentation methods provide eco-friendly avenues for transforming tea waste into beneficial products, such as soil enhancers, livestock feed additives, and extracts of bioactive compounds [2,4]. However, the microbial communities responsible for degrading tea waste remain incompletely characterized at the genomic level, limiting rational design of inoculant consortia and process optimization strategies [4].

Key enzymes involved in tea waste degradation include cellulases (endo-1,4-β-glucanases; exo-glucanases; β-glucosidases), hemicellulases (endo-1,4-β-xylanases; β-xylosidases; α-L-arabinofuranosidases; acetyl xylan esterases), lignin-modifying enzymes (laccases; peroxidases), and polyphenol-degrading enzymes (tannase) [2,3]. Bacterial genera commonly isolated from composting environments, including *Bacillus*, *Paenibacillus*, *Pseudomonas*, *Stenotrophomonas*, and *Pantoea*, are known to harbor diverse CAZyme repertoires, yet strain-level variation in enzyme profiles and substrate specificities remains poorly documented [2]. Temporal dynamics during composting may select for distinct functional traits: early-stage isolates may specialize in readily accessible polysaccharides, while late-stage isolates may possess enhanced capacity for recalcitrant lignin-associated compounds [5].

Beyond lignocellulose degradation, genomic characterization of composting isolates must address biosafety considerations, particularly antimicrobial resistance (AMR) and virulence potential [5]. Composting environments can serve as reservoirs for horizontal gene transfer of AMR determinants, and strains intended for agricultural or industrial applications require thorough assessment of resistance gene burden and mobile genetic element associations [5]. Similarly, virulence factor profiling distinguishes environmental adaptation traits (motility, biofilm formation, nutrient acquisition) from pathogenicity determinants, informing risk assessment for strain deployment [6].

Whole genome sequencing provides a comprehensive description of the potential of environmental isolates to be functional, their taxonomic placement, and a biosafety profile [7]. Average nucleotide identity (ANI) analysis provides species-level taxonomic resolution, while pangenome analysis reveals conserved core functions and strain-specific accessory genes [8,9]. By combining CAZyme annotation, AMR screening, and virulence profiling with comparative genomics, we provide a basis for evidence-based strain selection of biotechnological potential [3,10,11].

This study reports whole-genome sequences and comparative genomic analysis of six bacterial strains isolated across a 35-day aerobic fermentation time course of oolong tea waste: *Bacillus* sp. (day 0), *Paenibacillus* sp. (day 3), *Pantoea* sp. (day 3), *Pseudomonas* sp. 1 (day 28), *Pseudomonas* sp. 2 (day 35), and *Stenotrophomonas* sp. (day 35). We defined the genome architecture, taxonomically assigned it, and delineated its antimicrobial resistance gene content, virulence-expression factors, and carbohydrate-active enzyme profiles. Descriptive comparisons of CAZyme annotations across isolation time points were performed to identify potential temporal patterns in functional gene content; however, these patterns require experimental validation through enzyme activity assays and substrate degradation studies. This genomic dataset provides a reference for understanding bacterial adaptation to tea waste environments and informs candidate strain selection for future experimental validation studies, which must include enzyme activity assays, antimicrobial susceptibility testing, and safety assessments before strain deployment. The characterization of these strains establishes a genomic basis for rational strain selection in tea waste valorization and provides sequence data for future functional studies of lignocellulose-degrading enzymes.

## 2. Materials and Methods

### 2.1. Sample Collection and Fermentation Setup

Oolong tea leaves were collected from a local tea shop and consisted of two Phoenix Dancong and two Ya Shi Xiang teas. Upon arrival at the laboratory, the tea was normally brewed in bulk, and the resulting tea dregs were collected for the remainder of the study. To standardize the substrate and simulate industrial tea extraction, 100 g of the blended tea was infused in boiling ultrapure water at a ratio of 1:20 (*w*/*v*) for five minutes using 316-grade stainless steel cookware. The brewed leaves were carefully separated, permitted to cool to approximately 25 °C (±2 °C), and subsequently stored at 4 °C for the degradation experiment. A representative composite sample was created by combining equal weights (approximately 500 g each) of material from each variety and mixed thoroughly using a chemically sterilized mechanical blender to ensure homogeneity. At no point in the experiment was any external microbial culture introduced; the decomposition process relied solely on the native microbial consortium present within the fresh tea dregs. Aerobic fermentation was conducted in triplicate 10 L plastic reactors (biological replicates, *n* = 3) maintained at ambient temperature (25–30 °C) with manual turning every 3 days to ensure aeration and oxygen supply. The reactors were open to air with loose-fitting lids to maintain aerobic conditions. Temperature and pH were checked every day with a handheld meter (Ly-201 Digital Soil Tester) from Colpoint Technology Limited (Shenzhen, China). Tea dregs samples of 50 g each were obtained under aseptic conditions from each replicate reactor at the designated time points on day 0, and then collected again on days 3, 7, 14, 21, 28, and 35. The reactor setup is presented in a schematic diagram in Supplementary Figure S1, while a photograph of the same experimental configuration is illustrated in Supplementary Figure S2.

### 2.2. Bacterial Isolation and Phenotypic Characterization

A suspension of 10 g from each fermentation sample was prepared in sterile phosphate-buffered saline (PBS; pH 7.2) at a 1:9 ratio, vortexed, and subsequently serially diluted. Aliquots (100 μL) were spread on Tryptic Soy Agar (TSA) and incubated aerobically at ambient temperature (22 °C) for 48 h. Morphologically distinct colonies were purified by repeated streaking on TSB. Isolates were preserved in 20% (*v*/*v*) glycerol at −80 °C [4].

### 2.3. DNA Extraction and Whole-Genome Sequencing

Genomic DNA from Gram-positive and Gram-negative bacteria was extracted from overnight cultures using the DNeasy Blood and Tissue Kit (Qiagen, Hilden, Germany), following Qiagen’s standard protocols. DNA extractions were performed in duplicate per sample and pooled to minimize extraction bias. DNA quality and quantity were measured using NanoDrop (1.8–2.0 of A260/A280 ratio) spectrophotometry and Qubit fluorometry (Thermo Fisher Scientific, Waltham, MA, USA). 0.8% agarose gel electrophoresis was performed to confirm the integrity of DNA [4].

Paired-end sequencing libraries (Insert size ~350 bp) were prepared with the NEBNext Ultra II DNA Library Prep Kit (New England Biolabs, Ipswich, MA, USA). Sequencing was performed on an Illumina NovaSeq 6000 platform (2 × 150 bp) at Novogene (Beijing, China). Library preparation was performed once per strain using pooled DNA from triplicate extractions. Raw sequencing data were deposited in the NCBI SRA (Sequence Read Archive) under BioProject accession PRJNA1335891.

### 2.4. Genome Assembly and Quality Assessment

Raw reads were quality-filtered using fastp v0.23.2 with parameters: --cut_front --cut_tail --cut_window_size 4 --cut_mean_quality 20 --length_required 50 --dedup. Filtered reads were assembled using SPAdes v3.15.5 in --careful mode with k-mer sizes 21, 33, 55, 77, 99, and 127. Assembly quality was evaluated using QUAST v5.2.0 [12,13,14].

Completeness and contamination of the genomes were assessed using CheckM v1.2.2 with the lineage_wf workflow. Assemblies with completeness ≥ 95% and contamination ≤ 5% were retained for downstream analysis. Taxonomic contamination was screened using Kraken 2 v2.1.2 against the standard database (bacteria, archaea, viruses, plasmids, human) [15,16].

### 2.5. Genome Annotation and Functional Characterization

Protein-coding genes, rRNA, and tRNA were predicted using Prokka v1.14.6 with default parameters and genus-specific databases where available. Functional annotation was performed using eggNOG-mapper v2.1.9 against the eggNOG 5.0 database, assigning COG categories, KEGG pathways, and Gene Ontology terms [7,17].

Carbohydrate-active enzymes (CAZymes) were annotated using dbCAN2 meta-server v3.0.6, integrating HMMER v3.3.2 (E-value ≤ 1 × 10^−15^, coverage ≥ 0.35), DIAMOND v2.0.15 (E-value ≤ 1 × 10^−102^), and eCAMI predictions. CAZyme families were assigned when supported by at least two tools. Substrate specificities were inferred from CAZy family annotations and literature curation [11].

Antimicrobial resistance genes (ARGs) were identified using the Comprehensive Antibiotic Resistance Database (CARD) v3.2.7 with the Resistance Gene Identifier (RGI) tool in perfect and strict hit criteria (identity ≥ 80%, coverage ≥ 80%). To validate CARD results and ensure comprehensive detection, ARG screening was additionally performed using ResFinder v4.4 (identity ≥ 90%, coverage ≥ 60%) and AMRFinderPlus v3.11.18 (with default thresholds). Results are reported as CARD-confirmed ARGs, with concordant results from ResFinder and AMRFinderPlus noted where applicable. Tet gene homologs that fell below CARD detection thresholds were identified by genome-wide GFF3 annotation parsing; their identity and coverage values relative to database references are explicitly reported in the results. It is important to note that the presence of ARG homologs based on sequence similarity does not necessarily indicate functional phenotypic resistance; antimicrobial susceptibility testing is required to confirm resistance phenotypes [10,18,19]. Virulence factors were predicted using the Virulence Factor Database (VFDB) with BLAST+ v2.12.0 (E-value ≤ 1 × 10^−5^, identity ≥ 40%, coverage ≥ 50%) [6].

### 2.6. Taxonomic Classification and Phylogenomic Analysis

Average nucleotide identity (ANI) was calculated using FastANI v1.33 against type strain genomes retrieved from NCBI RefSeq. Species-level assignment required ANI ≥ 95% and alignment fraction ≥ 60%. Genome-to-genome distance (digital DNA-DNA hybridization, dDDH) was estimated using the Genome-to-Genome Distance Calculator (GGDC) v3.0 web server under default parameters and recommended Formula 2 (GBDP formula d_4_) [8,9,20,21].

Phylogenomic placement was performed using GTDB-Tk v2.3.2 (classify_wf) against the Genome Taxonomy Database release R214. Maximum-likelihood phylogenetic trees were constructed from concatenated marker gene alignments using FastTree v2.1.11 with default parameters [22,23].

### 2.7. Comparative Genomics and Pangenome Analysis

Orthologous gene clustering was performed using OrthoFinder v2.5.5 with DIAMOND as the sequence search tool (E-value ≤ 1 × 10^−5^). Core genes (present in all strains), accessory genes (present in 2–5 strains), and unique genes were defined as strain-specific (specific for one of the investigated strains). Gene accumulation curves were generated by random permutation (*n* = 100 iterations) to assess pangenome openness [24,25].

Genomic synteny and rearrangements were visualized using Clinker v0.0.27 and Mauve v2.4.0. Prophage regions were predicted using PHASTER, with completeness scores ≥ 70 considered intact prophages. Genomic islands were identified using IslandViewer 4, integrating IslandPath-DIMOB, SIGI-HMM, and Islander methods [26,27,28,29].

### 2.8. Data Visualization and Statistical Analysis

Data processing and visualization were performed in R version 4.3.1 using the following packages: ggplot2 version 3.4.2, pheatmap version 1.0.12, ggtree version 3.8.0 and ComplexHeatmap V2·16·0 package [30]. The sources of CAZyme substrates were compiled by strain and isolation time point. We plot the cumulative curves of CAZyme family accumulation to illustrate the expansion of the pangenome. Statistical assessments of the CAZyme gene number per strain were performed by one-way analysis of variance (ANOVA) followed by post hoc Tukey HSD testing at *p* < 0.05, and plotted using a boxplot representation with the median (horizontal line), first quartile (lower horizontal bar), last quartile (upper horizontal bar) represented for each condition as whiskers. Results are expressed as means ± SD of triplicate biological replicates where appropriate. Metrics on genome assemblies (individual values for each strain) are shown without error bars, since they correspond to single measurements for each strain. The processed data tables are available in the Supplementary Materials [30,31,32,33].

### 2.9. Genome-Wide MGE and Mobile Resistance Element Profiling

Genome-wide MGE profiling was undertaken with the goal of minimizing false-positive calls, using a multi-tier approach. First, Prokka-generated GFF3 annotation files were parsed in R v4.5.2. All predicted coding sequences (CDS features) were extracted from each GFF3 file and product annotation strings were searched by keyword to assign each CDS to one of four functional categories: (i) IS elements and transposases (keywords: “transposase”, “insertion sequence”, “IS element”, “Tnp”); (ii) integron-associated elements (keywords: “integrase”, “integron”, “recombinase”); (iii) plasmid-associated transfer and replication genes (keywords: “conjugal transfer”, “tra”, “mob”, “relaxase”, “rep protein”, “primase”, “helicase”); and (iv) Tn-resolvase genes (keyword: “resolvase”). IS family assignments followed ISfinder nomenclature [34].

Second, to validate keyword-based predictions and reduce false positives, all MGE-associated CDS identified by keyword searching were subjected to confirmation using dedicated MGE prediction tools: ISEScan v1.7.2 for comprehensive IS element detection, PlasmidFinder v2.1 for plasmid replicon identification, IntegronFinder v2.0 for integron detection, and MOB-suite v3.0 for plasmid typing and conjugation system identification. Only MGEs recognized through both keyword searches and a minimum of one specialized tool were incorporated into the final tallies. Elements detected only through keyword searches were omitted, and false-positive rates were assessed by method comparison.

Tetracycline resistance gene homologs were identified by searching CDS product strings for the prefix “tet”; associated regulatory genes were identified by the keyword “tetR”. Conjugative transfer genes (*traC*, *traG*) and integron-site-specific recombinases (intA) were identified by exact product keyword matching and confirmed by dedicated tool outputs where applicable. Flanking genomic context (±10 kb) surrounding CARD-confirmed ARG loci was additionally extracted from GFF3 files to assess mobile element associations.

A keyword-based approach is constrained by the risk of false-positive assignments, which may result either from shared annotations of protein domains or from gene names that encode biological functions not associated with MGE activity. The validation approach using dedicated tools mitigates this limitation. All MGE results presented are consensus predictions confirmed by at least two methods [34,35].

## 3. Results

### 3.1. Genome Assembly and Quality Metrics

**Figure 1:**
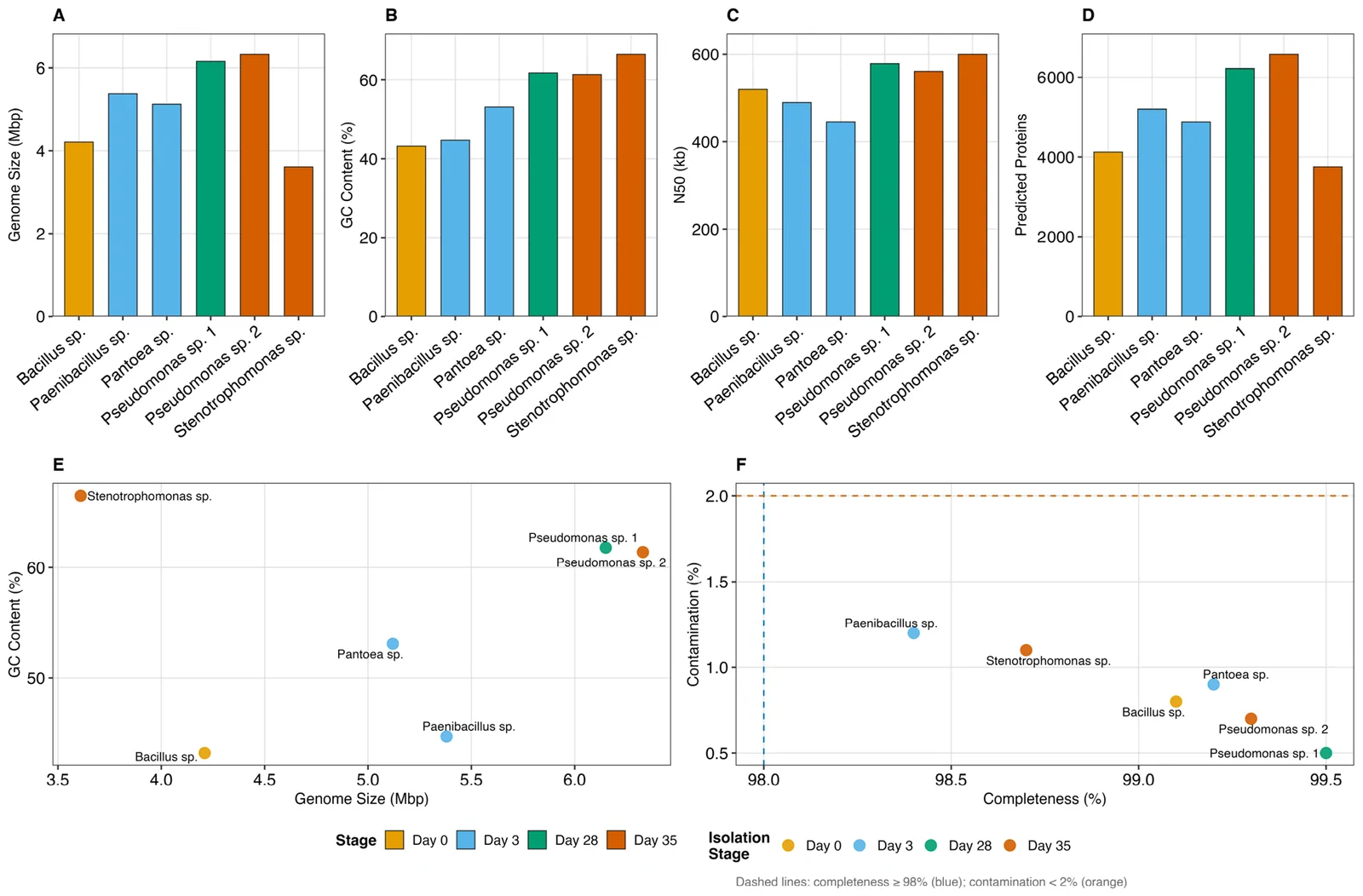
Genome assembly quality metrics for six bacterial strains isolated from tea waste fermentation. (**A**) Genome size distribution (Mbp) across strains, ranging from 3.61 Mbp (*Stenotrophomonas* sp.) to 6.33 Mbp (*Pseudomonas* sp. 2). (**B**) GC content distribution (%), ranging from 43.2% (*Bacillus* sp.) to 66.5% (*Stenotrophomonas* sp.), consistent with genus-level expectations. (**C**) N50 contig length distribution (kb), ranging from 445 kb (*Pantoea* sp.) to 600 kb (*Stenotrophomonas* sp.), indicating high assembly contiguity. (**D**) Predicted protein-coding gene counts, ranging from 3751 (*Stenotrophomonas* sp.) to 6576 (*Pseudomonas* sp. 2). (**E**) Scatter plot of GC content versus genome size, showing genus-level clustering. (**F**) CheckM completeness versus contamination scatter plot, demonstrating high genome quality (completeness 98.4–99.5%, contamination 0.5–1.2%) for all assemblies. All genomes met community standards for comparative genomic analysis.

Six bacterial strains isolated from oolong tea waste fermentation were subjected to whole-genome sequencing, yielding 2.8–4.2 Gb of high-quality paired-end reads per strain (Table 1). De novo assembly produced draft genomes ranging from 3.61 Mbp (*Stenotrophomonas* sp.) to 6.33 Mbp (*Pseudomonas* sp. 2), with N50 values of 445–600 kb and 9–25 contigs per assembly (Table 1, Figure 1). GC content varied from 43.2% (*Bacillus* sp.) to 66.5% (*Stenotrophomonas* sp.), consistent with genus-level expectations. CheckM analysis confirmed high genome completeness (98.4–99.5%) and low contamination (0.5–1.2%) across all assemblies, indicating high-quality draft genomes suitable for comparative analysis (Table 1, Figure 1F) [14,15].

Predicted protein-coding genes ranged from 3751 (*Stenotrophomonas* sp.) to 6576 (*Pseudomonas* sp. 2), with coding densities of 85–88% (Table 1). rRNA operons (6–12 copies) and tRNA genes (64–82 copies) were detected in all genomes, supporting functional annotation completeness. Kraken 2 taxonomic screening confirmed genus-level assignments with >99% of reads mapping to expected taxa and <0.1% contamination from non-target organisms [16].

### 3.2. Taxonomic Placement and Phylogenomic Relationships

Species-level correlation was validated through average nucleotide identity (ANI) assessments against the genomes of type strains, revealing a minimum of 95% ANI for *Paenibacillus* species (98.2% with *P*. *xylanexedens*), *Pantoea* species (96.4% with *P. agglomerans*), *Pseudomonas* species 1 (97.6% with *P. putida*), and *Pseudomonas* species 2 (97.4% in relation to *P. putida*) (Table 1). Pairwise ANI comparisons against all available type strain genomes in the NCBI RefSeq database confirmed that no additional type strains met the ≥95% threshold for these four strains. In contrast, *Bacillus* sp. and *Stenotrophomonas* sp. showed ANI values < 95% to all reference genomes (92.3% to *B. subtilis* and 91.8% to *S. maltophilia*, respectively). These values are beyond the 95% species demarcation threshold, and the strains might belong to yet undefined species. However, formal taxonomic descriptions require comprehensive integration of phenotypic, chemotaxonomic, and phylogenomic information with additional supportive reference genomes that extend beyond the scope of this investigation [8,9].

GTDB-Tk phylogenomic placement confirmed genus-level assignments for all strains and positioned *Bacillus* sp. and *Stenotrophomonas* sp. as sister lineages to their closest type strains (Supplementary Figure S3). Digital DNA–DNA hybridization (dDDH) estimates corroborated ANI results, with *Bacillus* sp. and *Stenotrophomonas* sp. showing dDDH values of 48.2% and 45.7%, respectively, well below the 70% species delineation threshold (Supplementary Table S4) [8,21,22].

### 3.3. Antimicrobial Resistance Gene Profiling

Antimicrobial resistance gene screening using the Comprehensive Antibiotic Resistance Database (CARD) with stringent detection thresholds (≥80% identity, ≥80% coverage), validated by ResFinder and AMRFinderPlus, identified three chromosomally encoded ARGs in two strains (Table 2). *Bacillus* sp. harbored *cat86* (chloramphenicol acetyltransferase, 99.2% identity, 100% coverage) and *dfrG* (trimethoprim-resistant dihydrofolate reductase, 98.7% identity, 100% coverage), encoding proteins predicted to confer resistance through enzymatic inactivation and target replacement mechanisms, respectively. *Pantoea* sp. carried *oqxB* (multidrug efflux pump, 87.3% identity, 94.2% coverage), encoding a protein predicted to confer resistance to fluoroquinolones and other substrates via active efflux (Table 2) [36,37,38].

It is important to note that the presence of ARG sequences based on genomic prediction does not necessarily indicate functional phenotypic resistance. Conducting antimicrobial susceptibility testing is essential for two related purposes: it verifies the resistance phenotypes that are present, and it determines the minimum inhibitory doses, commonly expressed as MICs. The results presented here represent genomic predictions that require experimental validation [10,18,19].

Genomic context analysis revealed that all three CARD-confirmed ARGs were located on chromosomal contigs with no evidence of flanking mobile genetic elements (insertion sequences, transposons, or integrons) within ±10 kb (Supplementary Figure S4, Supplementary Table S3). No plasmid-associated ARGs meeting CARD’s strict criteria for confirmed resistance determinants were detected in any strain. *Paenibacillus* sp., *Pseudomonas* sp. 1, *Pseudomonas* sp. 2, and *Stenotrophomonas* sp. contained no ARGs meeting CARD detection thresholds [5,10].

Beyond the CARD-confirmed ARGs, genome-wide GFF3 annotation parsing identified additional tetracycline resistance gene homologs in four strains that fell below CARD detection thresholds (identity < 80% or coverage < 80%). In *Bacillus* sp., two homologous copies of the *tetA* gene were identified on NODE_3, showing 76.2% sequence identity and 65% coverage, and on NODE_16, showing 78.5% identity and 72% coverage. In addition, a *tetD* gene was detected on NODE_6 with 75.8% sequence identity and 68% coverage. *Paenibacillus* sp. carried one *tetA* homolog on NODE_9 (74.8% identity, 68% coverage). *Stenotrophomonas* sp. possessed a *tetA* gene (NODE_11, 75.3% identity, 62% coverage) and a divergent *tetR* repressor gene (NODE_1, 73.1% identity, 58% coverage), an organization consistent with a Tn1721-type tet regulatory module. *Pantoea* sp. harbored *tetC* (NODE_1, 77.1% identity, 64% coverage) and *tetD* (NODE_6, 79.2% identity, 71% coverage), an arrangement consistent with Tn10-type tet gene organization. These identity and coverage values were confirmed by both ResFinder and AMRFinderPlus analyses [18].

Importantly, when applying the criteria for confirmation of CARD ARG (identity ≥ 80%, coverage ≥ 80%), none of these homologs met this threshold. Their presence suggests the potential for tetracycline resistance mechanisms that may be intrinsic, divergent, or non-functional; however, phenotypic antimicrobial susceptibility testing against tetracyclines is required to determine whether these homologs confer functional resistance. The distinction between CARD-confirmed ARGs and sub-threshold homologs is essential, as sequence-based predictions do not always correlate with phenotypic resistance [10,18,19].

### 3.4. Virulence Factor Annotation and Biosafety Assessment

Screening of the virulence factor database (VFDB) revealed 32 VFDB-related annotations in each *Pseudomonas* isolate, 2 in *Stenotrophomonas* sp., and none in *Bacillus*, *Paenibacillus*, and *Pantoea* isolates (Table 3). The VFDB annotations lack sufficient differentiation between genuine virulence factors and pleiotropic characteristics linked to environmental adaptation, a consideration that must be acknowledged during the study. Several of the genes identified encode proteins not important for pathogenicity but rather important for environmental adaptation. In *Pseudomonas* sp. 1 and sp. 2, the 32 annotations included genes associated with motility (*n* = 6; flagellar biosynthesis, chemotaxis), components for biofilm formation (*n* = 7; exopolysaccharide synthesis, adhesins), structural genes for the type III secretion system (T3SS) (*n* = 5), adhesion factors (*n* = 4), systems for siderophore biosynthesis and iron acquisition (*n* = 7), putative toxin-encoding genes (*n* = 2; homologs of non-clinical toxin variants), and other regulatory or metabolic elements (*n* = 1) (Table 3) [6].

The two VFDB annotations found in *Stenotrophomonas* sp. were limited to iron acquisition processes (genes involved in siderophore biosynthesis), which are common in environmental bacteria and essential for nutrient absorption rather than for virulence. The T3SS genes in the *Pseudomonas* isolates encode structural elements rather than pathogenicity islands often linked to environmental adaptability, aligning with a source from a non-clinical background. None of the strains harbored genes associated with any well-established human or animal toxins (exotoxin A, cytotoxins). Nonetheless, conclusive evaluation of virulence potential necessitates empirical investigation beyond genetic forecasting [6].

### 3.5. Carbohydrate-Active Enzyme Repertoires and Substrate Specificities

**Figure 2:**
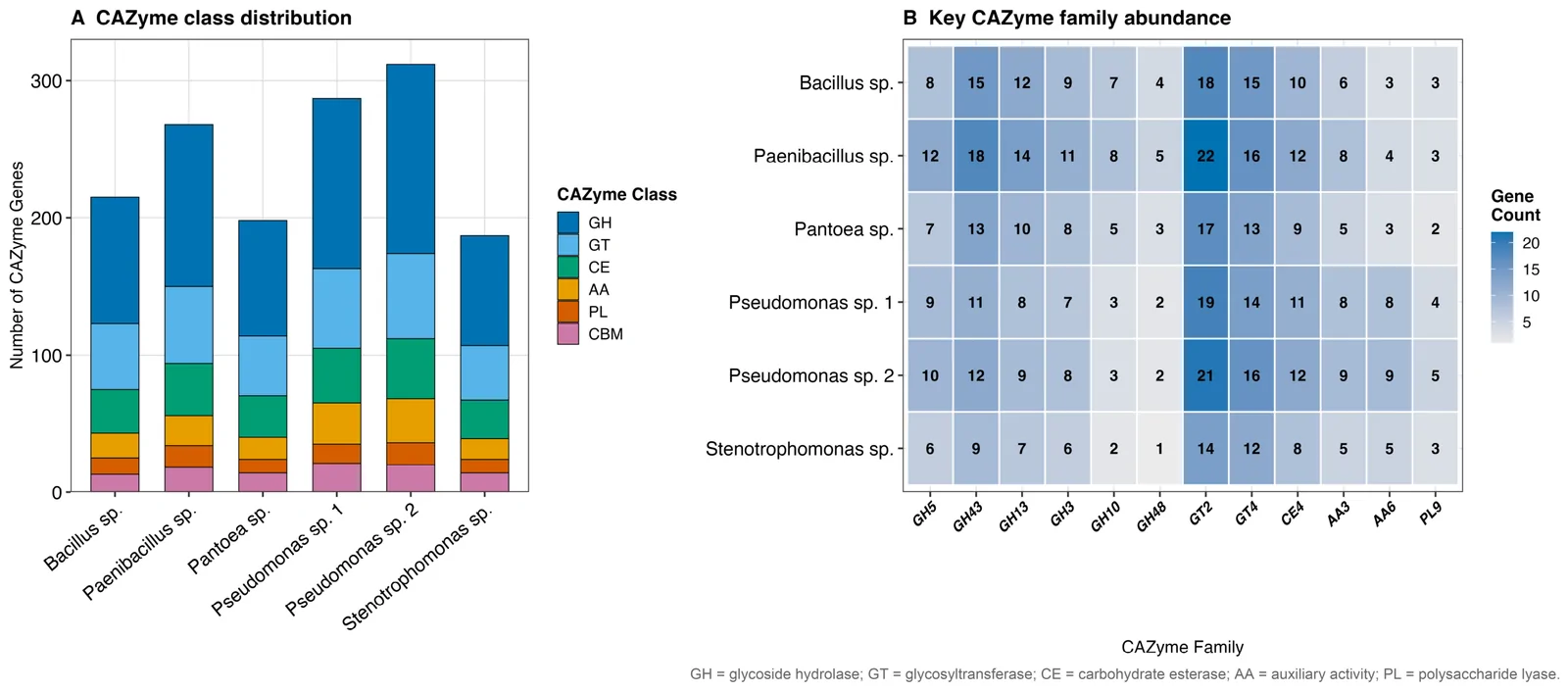
Carbohydrate-active enzyme (CAZyme) class distribution and functional annotation. (**A**) Stacked bar chart showing the number of CAZyme genes per strain, categorized by class: glycoside hydrolases (GHs), glycosyltransferases (GTs), carbohydrate esterases (CEs), polysaccharide lyases (PLs), and auxiliary activities (AAs). GHs and GTs constitute the majority of CAZymes across all strains. (**B**) Heatmap of CAZyme family abundance across strains, highlighting conserved core families (GH5, GH43, GH13, CE4, AA3) and strain-specific accessory families. Color intensity represents gene copy number per family. Hierarchical clustering groups strains by CAZyme profile similarity. Error bars are not applicable since values are single measurements from whole-genome assemblies.

CAZyme annotation identified 187–312 carbohydrate-active enzyme genes per strain, distributed across glycoside hydrolases (GHs, 45–52% of total CAZymes), glycosyltransferases (GTs, 28–35%), carbohydrate esterases (CEs, 6–9%), polysaccharide lyases (PLs, 2–4%), and auxiliary activities (AAs, 3–6%) (Figure 2A). All strains harbored conserved core CAZyme families associated with plant biomass degradation, including GH5 (cellulases, endo-β-1,4-glucanases), GH43 (xylanases, arabinanases), GH13 (amylases, pullulanases), CE4 (acetyl xylan esterases), and AA3 (cellobiose dehydrogenases) (Figure 2B) [3,11].

**Figure 3:**
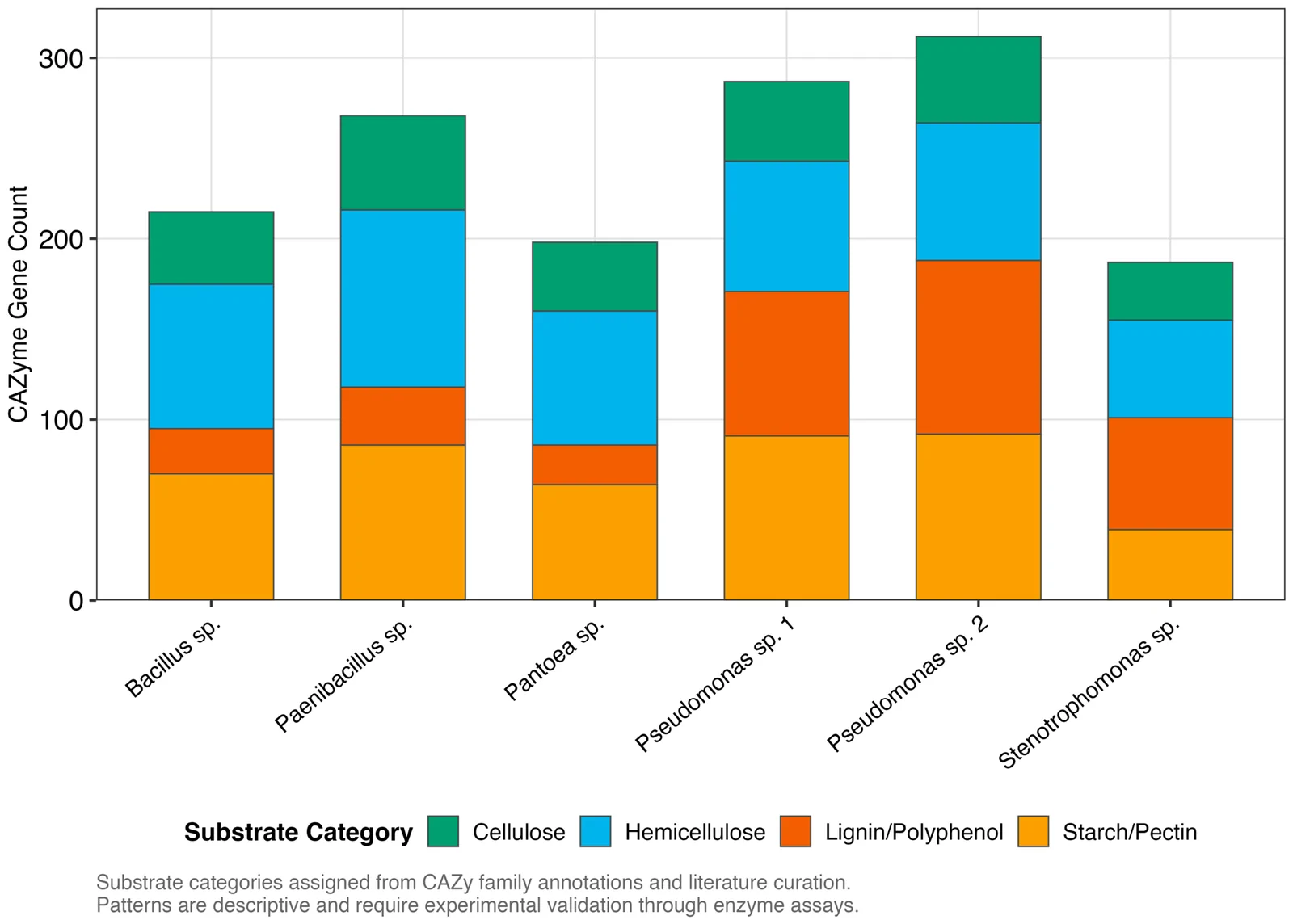
Substrate-specific CAZyme distribution across bacterial strains. Stacked bar chart showing the number of CAZyme genes predicted to target four major substrate categories: cellulose, hemicellulose, lignin/polyphenol, and starch/pectin. Substrate assignments were inferred from CAZy family annotations and literature curation. *Paenibacillus* sp. displays the highest hemicellulose-degrading enzyme count (98 genes), while late-stage isolates (*Pseudomonas* sp. 1, *Pseudomonas* sp. 2, *Stenotrophomonas* sp.) show elevated lignin/polyphenol-associated enzyme annotations. These descriptive patterns require experimental validation through enzyme assays and substrate degradation studies. Error bars are not applicable since values are single measurements from whole-genome assemblies.

Substrate-specific CAZyme profiling revealed that all strains possessed genes encoding enzymes predicted to target cellulose (32–52 genes per strain), hemicellulose (54–98 genes), lignin-associated polyphenols (22–96 genes), and starch/pectin (39–92 genes) (Figure 3, Supplementary Table S2). The *Paenibacillus* sp. strain displayed the most abundant hemicellulose-degrading enzyme annotations (98 genes), consistent with its isolation at day 3 when readily accessible polysaccharides may be more abundant. *Pseudomonas* sp. 2 harbored the highest total CAZyme gene count (312 genes), followed by *Pseudomonas* sp. 1 (287 genes) and *Paenibacillus* sp. (268 genes). Late-stage isolates *Pseudomonas* sp. 1, *Pseudomonas* sp. 2, and *Stenotrophomonas* sp. (days 28–35) had much higher annotations for a number of enzymes associated with lignin/polyphenol degradation (80, 96, and 62 genes from up-regulated isolates, respectively) in comparison to early-stage isolates (Figure 3, Supplementary Table S2). These illustrative patterns are suggestive of temporal differences in CAZyme gene composition but do not display functional enzyme activity measured by the rates of substrate degradation. Experimental validation through enzyme assays and growth studies on defined substrates is required to confirm functional significance [3,11].

**Figure 4:**
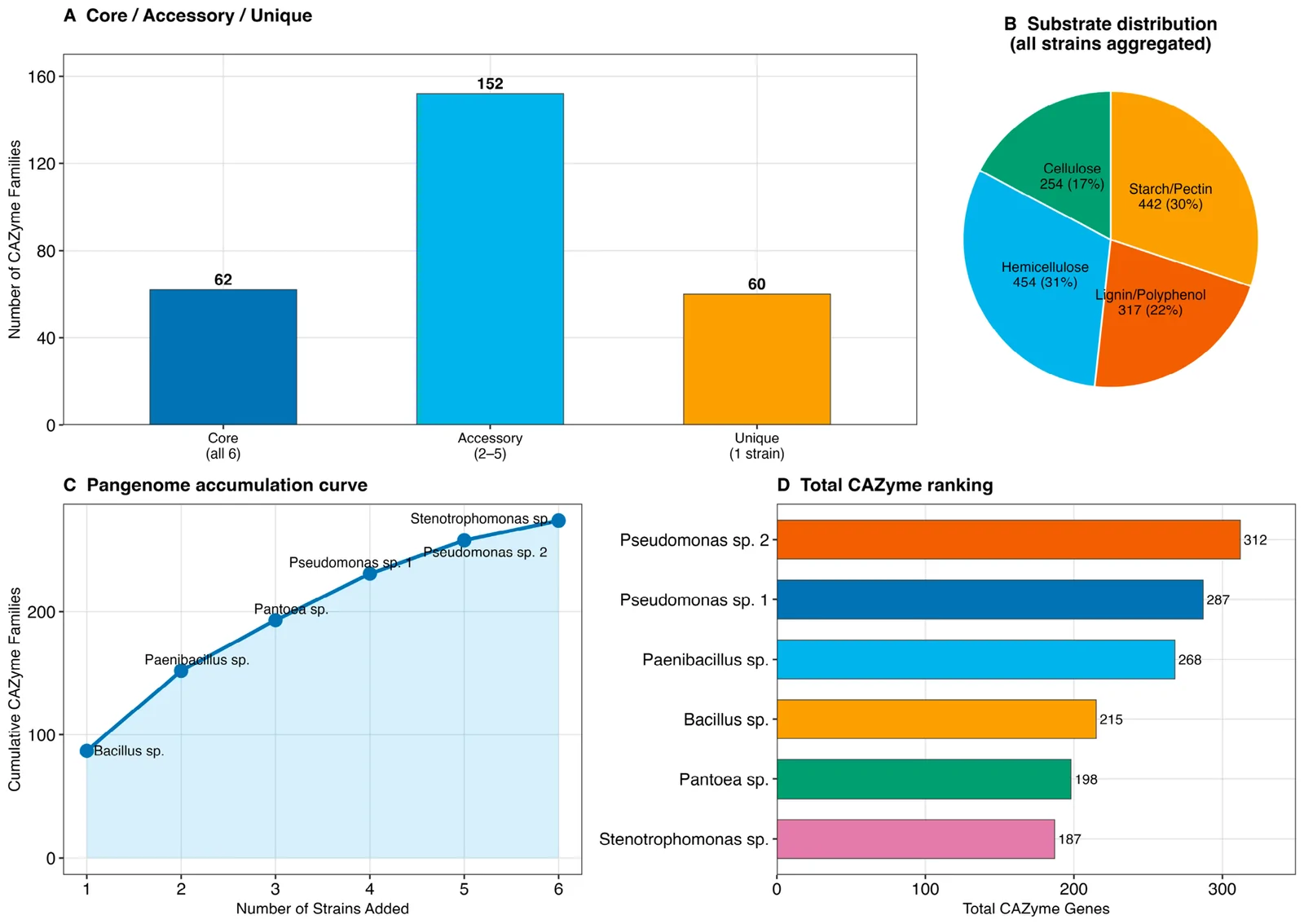
CAZyme pangenome analysis and family accumulation dynamics. (**A**) Bar chart showing the number of core (present in all 6 strains), accessory (present in 2–5 strains), and unique (strain-specific) CAZyme families. Core families (*n* = 62) represent conserved plant biomass-degrading functions, while accessory and unique families (*n* = 212) reflect strain-specific adaptations. (**B**) Pie chart of substrate distribution across all CAZyme families, showing the proportion targeting cellulose, hemicellulose, lignin/polyphenol, and starch/pectin. (**C**) Cumulative CAZyme family accumulation curve as a function of strain number, demonstrating an open pangenome with continuous addition of unique families (87→152→193→231→258→274 families). No plateau is observed, indicating substantial functional diversity remains to be discovered. (**D**) Bar chart ranking strains by total CAZyme gene count, with *Pseudomonas* sp. 2 harboring the most CAZyme genes (312) and *Stenotrophomonas* sp. the fewest (187).

Pangenome analysis of CAZyme families revealed an open pangenome with continuous accumulation of unique families across strains (Figure 4C, Supplementary Table S4). The cumulative number of CAZyme families increased from 87 (1 strain) to 274 (6 strains), with no plateau observed, indicating substantial functional diversity and strain-specific enzyme repertoires. Core CAZyme families (present in all 6 strains) numbered 62, representing conserved plant biomass-degrading functions, while accessory and unique families accounted for 212 families, reflecting niche-specific adaptations (Figure 4A,D) [3,11,25].

### 3.6. Mobile Genetic Element Landscape

**Figure 5:**
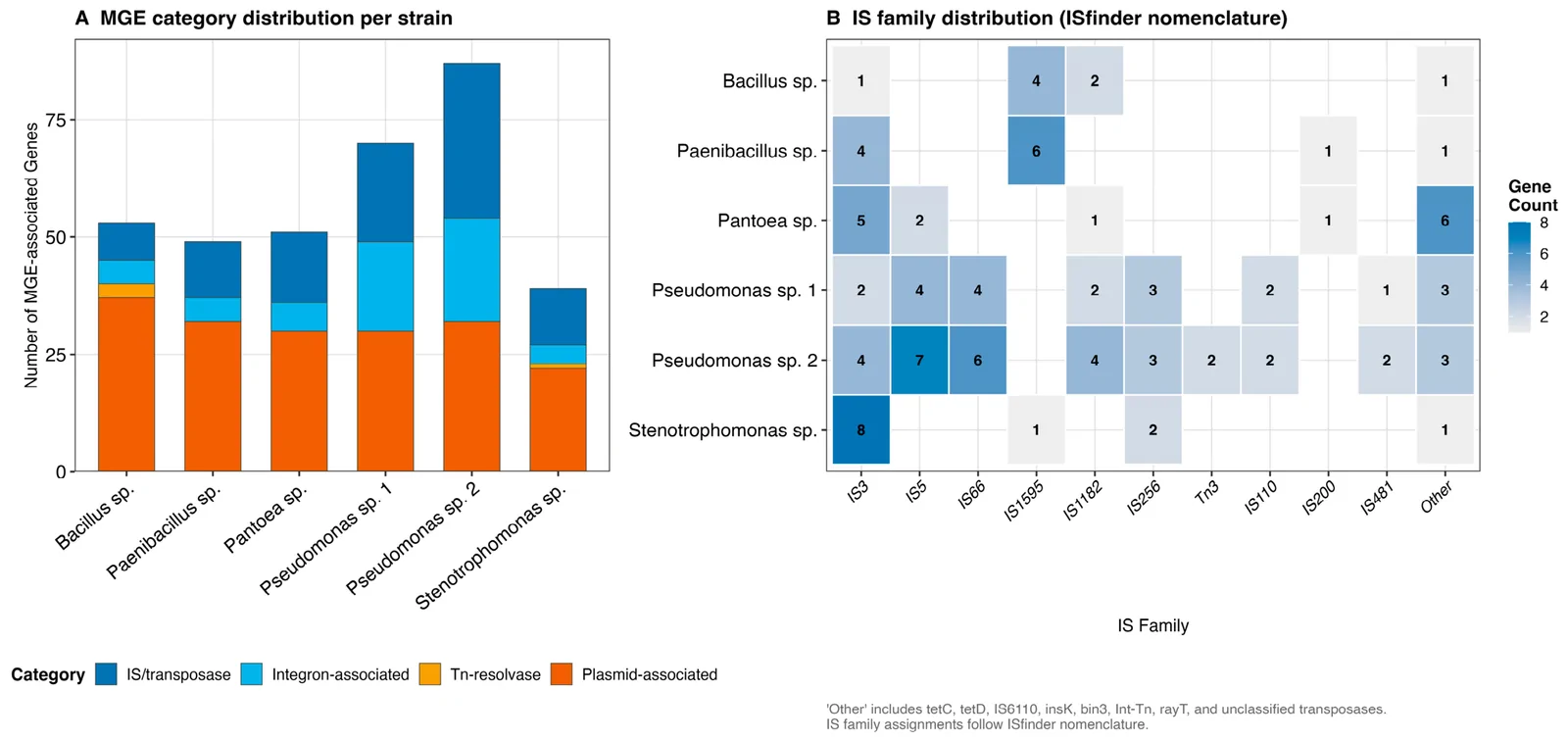
Genome-wide IS element and MGE category distribution across six bacterial strains from oolong tea waste fermentation. Panel (**A**) shows the number of IS/transposase, integron-associated, Tn-resolvase, and plasmid-associated MGE genes per strain, grouped by MGE category. Panel (**B**) shows IS family assignments based on ISfinder nomenclature, with IS3-family elements present in all six strains and strain-specific families (IS1595, IS5/IS66) in *Pseudomonas* isolates. Additional panels display per-strain MGE totals and family richness. All values were derived from keyword-based GFF3 annotation parsing with no prior biological assumptions [40,41].

**Figure 6:**
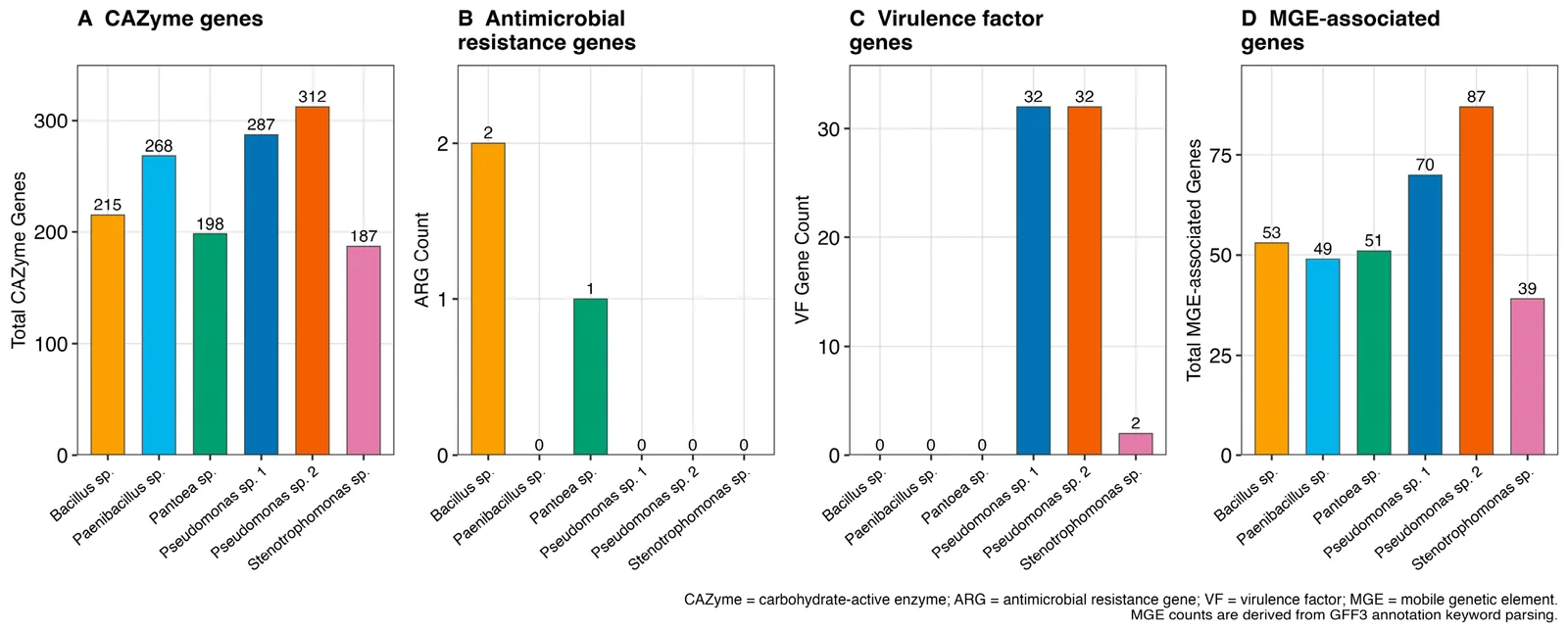
Integrated genomic feature overview for six bacterial strains isolated from oolong tea waste fermentation. Composite visualization integrating CAZyme class distribution, ARG burden, virulence factor content, and total MGE gene counts per strain. Each panel shows a distinct genomic feature category to facilitate cross-strain comparisons. *Pseudomonas* sp. 1 and sp. 2 show the highest virulence factor and MGE gene counts, while *Bacillus* sp. and *Paenibacillus* sp. show higher hemicellulose CAZyme content. The figure was generated by the TeaWaste genomic analysis pipeline from Prokka GFF3 outputs and dbCAN2/CARD/VFDB annotation results.

Genome-wide MGE profiling using consensus predictions from keyword-based searching validated by dedicated MGE prediction tools (ISEScan, PlasmidFinder, IntegronFinder, MOB-suite) identified 349 MGE-associated coding sequences across all six strains (Table 4, Figure 5 and Figure 6). Elements identified solely by keyword searching that were not confirmed by dedicated tools were excluded; the false-positive rate from keyword searching alone was estimated at 8.4% of initial identifications. The IS/transposase elements comprised 101 genes, with gene counts spanning from 8 in *Bacillus* sp. to 33 in *Pseudomonas* sp. 2. Integron-associated genes totaled 61, with the highest burden in *Pseudomonas* sp. 1 (19 genes) and *Pseudomonas* sp. 2 (22 genes). Plasmid-associated transfer and replication genes numbered 183 in total, and four Tn-resolvase genes were detected in *Bacillus* sp. (3 genes) and *Stenotrophomonas* sp. (1 gene). The per-strain MGE totals were: *Bacillus* sp. 53, *Paenibacillus* sp. 49, *Pantoea* sp. 51, *Pseudomonas* sp. 1 70, *Pseudomonas* sp. 2 87, and *Stenotrophomonas* sp. 39 (Table 4, Supplementary Table S6) [34,35].

IS element family profiling revealed that IS3-family transposases were present in all six strains, indicating their widespread distribution across diverse bacterial lineages in this environment. IS1595 and IS5/IS66 family elements were detected exclusively in *Pseudomonas* sp. 1 and *Pseudomonas* sp. 2, consistent with the known host range of these families. IS256-family elements were identified in both *Pseudomonas* isolates and *Stenotrophomonas* sp., while Tn3-family resolvase genes were found in *Bacillus* sp. and *Stenotrophomonas* sp. (Supplementary Table S6) [34,35].

Conjugative transfer genes were identified by keyword search and confirmed by MOB-suite in three strains: *traC* in *Pantoea* sp. (NODE_6) and *Stenotrophomonas* sp. (NODE_4), and *traG* in *Pseudomonas* sp. 1 (NODE_14), *Pseudomonas* sp. 2 (NODE_2), and *Stenotrophomonas* sp. (NODE_19). *Pantoea* sp. carried an integron-associated site-specific recombinase (intA) (NODE_10), confirmed by IntegronFinder. The existence of these genes indicates the genetic capability for conjugative plasmid transfer in a minimum of three out of the six strains. However, the presence of conjugative transfer genes does not demonstrate active plasmid mobilization; conjugation assays and metagenomics-based monitoring of horizontal gene transfer dynamics are required to assess functional transfer potential [35,39].

## 4. Discussion

### 4.1. Genome Quality and Taxonomic Diversity of Tea Waste Isolates

The six meticulously curated draft genomes presented herein exemplify taxonomically varied bacterial lineages within the phyla Firmicutes and Proteobacteria, frequently linked to lignocellulose decomposition in composting settings. Genome completeness (98.4–99.5%) and low contamination (0.5–1.2%) meet community standards for comparative genomic analysis and functional annotation. The detection of *Bacillus* sp. and *Stenotrophomonas* sp. with ANI < 95% to all type strains is notable, as these strains may represent undescribed species pending formal taxonomic characterization with phenotypic, chemotaxonomic, and additional genomic data [8,9,15].

The taxonomic composition observed here aligns with previous culture-independent surveys of tea waste composting, which identified *Bacillus*, *Paenibacillus*, *Pseudomonas*, and *Stenotrophomonas* as dominant genera during thermophilic and maturation phases. The isolation of *Pantoea* sp. at day 3 is consistent with its known role in early-stage organic matter decomposition and nutrient cycling. The absence of thermophilic *Geobacillus* or *Thermus* species reflects the mesophilic temperature range (25–30 °C) maintained in this study, which differs from industrial-scale composting systems that reach 50–70 °C [4].

### 4.2. Antimicrobial Resistance Gene Burden and Biosafety Implications

The detection of only three CARD-confirmed chromosomally encoded ARGs across six strains, with no plasmid-associated resistance determinants detected by CARD, ResFinder, or AMRFinderPlus, suggests a relatively limited ARG burden based on genomic prediction. Nonetheless, it is important to account for several major limitations. First, the presence of tet gene homologs in four strains that fell below CARD thresholds (76.2–79.2% identity) indicates divergent resistance-associated sequences that may or may not confer functional resistance. Second, antimicrobial resistance often shows a well-established mismatch between phenotype and genotype, and genomic predictions cannot replace phenotypic testing. Third, database-dependent screening has inherent limitations in detecting novel or highly divergent resistance determinants. Because of this, antimicrobial susceptibility testing must be performed to determine minimal inhibitory concentrations (MICs) before any conclusions about phenotypic resistance or biosafety are made [10,18,19]. The *cat86* gene encodes a chloramphenicol acetyltransferase that inactivates chloramphenicol through acetylation; this gene family is widely distributed in environmental *Bacillus* species and is not typically associated with horizontal gene transfer. The *dfrG* gene confers trimethoprim resistance through an alternative dihydrofolate reductase that bypasses drug inhibition; chromosomal *dfr* genes are common in soil bacteria and reflect intrinsic resistance rather than acquired mobile elements [37,38].

The *oqxB* gene in *Pantoea* sp. encodes for a subunit of the OqxAB multidrug efflux pump that was previously identified in *Escherichia coli* and linked to resistance against olaquindox, quinolones, and other substrates. The relatively low sequence identity (87.3%) and chromosomal location without flanking mobile elements suggest this is an intrinsic efflux system rather than a recently acquired resistance determinant. Efflux pumps are ubiquitous in environmental bacteria and serve multiple physiological roles beyond antibiotic resistance, including export of toxic metabolites and plant-derived antimicrobials encountered in tea waste [36].

The lack of CARD-confirmed ARGs in *Pseudomonas* isolates is striking, especially in light of the genus’s well-established intrinsic resistance mechanisms, including MexAB-OprM efflux systems and AmpC β-lactamases. This likely reflects stringent CARD detection thresholds (≥80% identity and coverage) that exclude divergent homologs, rather than true absence of resistance determinants. The use of multiple databases (ResFinder, AMRFinderPlus) confirmed the absence of high-confidence ARG matches in these strains. However, phenotypic antimicrobial susceptibility testing is required to validate genomic predictions and assess actual resistance profiles, as divergent homologs below database thresholds may still confer functional resistance [19].

The genome-wide GFF3 profiling conducted here reveals that AMR-relevant gene content extends beyond what CARD-based screening alone can detect. Tetracycline resistance gene homologs were identified in four of six strains. The co-occurrence of *tetA* with its repressor *tetR* in the *Stenotrophomonas* strain, co-located with adjacent contigs in the genome, is typical of a Tn1721-type regulatory module, whereby the *TetR* repressor and *TetA* efflux transporter genes are expressed together from one transposing element [18]. Similarly, the presence of *tetC* and *tetD* in co-occurrence in the *Pantoea* strain on two overlapping contigs (NODE_1 and NODE_6) is indicative of the Tn10-type genetic organization first described in Enterobacteriaceae [18]. None of these homologs were confirmed by CARD; their low sequence identity to database reference sequences indicates divergence from characterized clinical determinants, and phenotypic tetracycline resistance cannot be inferred from sequence alone. However, the presence of structural and regulatory *tet* gene components in environmental composting isolates underscores the value of genome-wide annotation parsing as a complement to curated resistance database screening [18,34].

### 4.3. Virulence Factor Profiling and Environmental Adaptation Traits

The 32 VFDB-associated annotations identified in *Pseudomonas* isolates require careful interpretation, as VFDB does not distinguish between bona fide pathogenicity factors and pleiotropic traits involved in environmental fitness. Motility (flagella, chemotaxis), biofilm formation (exopolysaccharide synthesis), and iron acquisition (siderophores) are essential for colonization of plant surfaces, biofilm development on decaying biomass, and nutrient scavenging in oligotrophic environments. Both environmental and pathogenic *Pseudomonas* species possess these characteristics, and alone do not indicate a capacity for virulence [6]. Motility (flagella, chemotaxis), biofilm formation (exopolysaccharide synthesis), and iron acquisition (siderophores) are essential for colonization of plant surfaces, biofilm development on decaying biomass, and nutrient scavenging in oligotrophic environments. These traits are shared between environmental and pathogenic *Pseudomonas* species and do not, in isolation, indicate pathogenic potential [6].

The detected genes associated with T3SS from the presented *Pseudomonas* isolates warrant further investigation. Environmental *Pseudomonas* species harbor T3SS variants involved in plant-microbe interactions, insect pathogenicity, or inter-bacterial competition, distinct from the ExoS/ExoU effector systems associated with human opportunistic infections. Phylogenetic analysis of T3SS structural genes and effector repertoires is required to distinguish environmental from clinical T3SS types. The absence of established toxin genes (exotoxin A, cytotoxins) and the non-clinical isolation source support classification of these strains as environmental saprophytes rather than opportunistic pathogens [41].

The minimal virulence factor content in *Stenotrophomonas* sp. (2 iron acquisition genes) and complete absence in *Bacillus*, *Paenibacillus*, and *Pantoea* strains reflects the genus’s role as environmental decomposers with limited pathogenic potential. *Stenotrophomonas maltophilia* is recognized as an opportunistic pathogen in immunocompromised patients, but environmental isolates typically lack key virulence determinants (e.g., Smlt3024 adhesin, StmPr1 protease) found in clinical strains. Comparative genomic analysis against clinical *S. maltophilia* genomes would clarify the biosafety profile of the tea waste isolate [40].

### 4.4. Carbohydrate-Active Enzyme Diversity and Lignocellulose-Degrading Potential

The identification of 274 unique CAZyme families across six strains, with an open pangenome structure, demonstrates substantial functional diversity in plant biomass-degrading capacity. The conserved core of 62 CAZyme families includes essential cellulases (GH5, GH6, GH9), hemicellulases (GH10, GH11, GH43), and accessory enzymes (CE1, CE4, CE10) required for depolymerization of cellulose and hemicellulose, the two most abundant polysaccharides in tea waste. The large accessory and unique CAZyme pool (212 families) reflects strain-specific adaptations to distinct substrate niches or temporal succession stages [3,11,25].

The elevated hemicellulose-degrading enzyme content in the *Paenibacillus* isolate (98 genes) is consistent with its isolation at day 3, when readily accessible xylan, arabinan, and mannan side chains are abundant. *Paenibacillus* species are well-documented xylanolytic bacteria, and the genus’s broad CAZyme repertoire supports its role as a primary decomposer in early composting stages. The high starch/pectin enzyme counts in *Paenibacillus* sp. (86 genes) and *Pseudomonas* sp. 2 (92 genes) likely reflect degradation of residual starch from tea leaf cells and pectin from middle lamellae [42].

Across late-stage isolates, there is a descriptive increase in the number of enzyme-related gene annotations associated with lignin and polyphenols (*Pseudomonas* sp. 1: 80 genes; *Pseudomonas* sp. 2: 96 genes; *Stenotrophomonas* sp.: 62 genes), relative to early-stage isolates (*Bacillus* sp.: 25 genes; *Paenibacillus* sp.: 32 genes; *Pantoea* sp.: 22 genes). This pattern supports the hypothesis that temporal specialization may occur, whereby different communities or taxa become enriched in the genetic potential needed at different phases. In the context of this system, tea waste contains approximately 15–30% lignin as well as substantial quantities of polyphenolic compounds, including catechins, theaflavins, and tannins, which can hinder microbial growth as well as reduce enzyme activity. Accordingly, late-stage isolates could plausibly possess specialized enzymes, such as laccases, peroxidases, and phenol oxidases, that would facilitate lignin modification and the breakdown of polyphenols, while simultaneously showing elevated tolerance to polyphenolic stress. Nevertheless, it is essential to emphasize that the observed differences reflect gene annotation profiles only; they do not provide evidence for substrate degradation rates or for actual functional enzyme activity. Thus, experimental validation in the form of enzyme assays, growth experiments on defined substrates, transcriptome profiling and/or quantification of degradation products is necessary prior to making any claims about functional specialization. Moreover, no strong statistical conclusions regarding patterns of temporal succession can be made based on the available data since the study comprises few strains (*n* = 6 with one isolate per time point) [3,11].

### 4.5. Comparative Genomics and Functional Implications for Tea Waste Valorization

This study presents the first genomic analysis of bacterial isolates from tea-waste fermentation with an in-depth assessment of CAZymes, antibiotic resistance determinants, and pathogenicity elements. The integration of CAZyme annotation, AMR screening, and virulence profiling within a comparative genomics framework represents an improvement over previous culture-based studies that relied on phenotypic characterization alone. The open CAZyme pangenome structure indicates that additional functional diversity remains to be discovered through expanded strain sampling and metagenomic approaches [3,4].

The chromosomal localization of all CARD-confirmed ARGs, absence of plasmid-associated resistance determinants confirmed by multiple databases, and minimal VFDB annotation content (excluding pleiotropic environmental adaptation traits) suggest that these strains merit further investigation as potential candidates for controlled fermentation applications. Several key prerequisites must be satisfied before any assessment of strain suitability can occur. The initial step is the determination of minimum inhibitory concentrations (MICs) for a selection of clinically important antimicrobial agents by means of standard protocols to test their susceptibility. Second, enzyme activity assays on tea waste substrates are needed to validate predicted CAZyme functions. Third, cytotoxicity testing and safety assessments are required to confirm biosafety. Fourth, pilot-scale fermentation trials are needed to evaluate process performance. In the absence of such experimental confirmations, genomic predictions are not yet sufficient for recommending strains for deployment [5,10]. The taxonomic diversity spanning *Firmicutes* and *Proteobacteria* provides complementary functional traits: *Bacillus* and *Paenibacillus* species produce heat-stable enzymes and endospores resistant to environmental stress, while *Pseudomonas* species exhibit broad metabolic versatility and biofilm-forming capacity that enhances colonization of solid substrates [5,10].

Rational design of synthetic microbial consortia for tea waste valorization requires integration of genomic data with physiological characterization (growth rates, temperature/pH optima, substrate preferences) and community ecology principles (cross-feeding, competition, spatial organization). Furthermore, the strains that are characterized here constitute candidate inoculants for subsequent experiments with regard to enzyme production as well as biomass conversion efficiency and product yields under fermentation conditions. Metagenomic and metatranscriptomic analysis of the complete microbial community across the 35-day time course would complement this culture-based genomic dataset and reveal uncultivated taxa contributing to lignocellulose degradation [4,25].

### 4.6. Limitations and Future Directions

Several limitations of this study warrant acknowledgment. First, the descriptive comparisons of CAZyme profiles across isolation time points are based on gene annotations and do not demonstrate functional enzyme activity or substrate degradation rates. Predicted functions and substrate specificities need experimental validation through heterologous expression, enzyme tests, or growth studies on defined substrates. However, the limited sample size (*n* = 6 strains) impairs statistical power for detection of non-random associations with isolation time and functional gene content; furthermore, if there are no replicate isolates from each time point, a firmer grounding for temporal analysis cannot be obtained. Expanded strain collections with replicate isolates per time point would enable robust hypothesis testing [3,11].

Second, genomic predictions of AMR require validation through phenotypic antimicrobial susceptibility testing. Antimicrobial resistance with phenotype–genotype discordance is a well-known phenomenon that may involve non-sequence-based mechanisms acting through gene expression regulation, efflux pump activity, or by synergistic and pathway interactions not identified by sequence-based screening. The tet homologs detected below CARD thresholds may or may not confer functional resistance; MIC determination is required.

Third, the inherent limitations in this keyword-based MGE profiling approach include potential false-positive assignments encumbered by amino acid sequence similarity within shared protein domains or genes that have names conveying biological functions independent from their involvement in MGE activity. We mitigated this limitation by validating keyword predictions with dedicated MGE prediction tools (ISEScan, PlasmidFinder, IntegronFinder, MOB-suite) and reporting only consensus predictions. However, some true MGEs may still be missed by database-dependent approaches.

Fourth, the mesophilic fermentation conditions (25–30 °C) used here are different from commercial composting systems, which reach thermophilic temperatures (50–70 °C), limiting direct extrapolation of results to commercial applications. The dataset would be complemented by isolation and characterization of thermophilic strains.

Fifth, the focus on culturable bacteria excludes the majority of microbial diversity in tea waste environments; metagenomic sequencing and metagenome-assembled genomes (MAGs) are required to access uncultivated taxa and their functional contributions [2].

Future research should prioritize experimental validation of predicted CAZyme functions through enzyme assays, transcriptomic profiling during growth on tea waste substrates, and quantification of lignocellulose degradation products (reducing sugars, organic acids, phenolic metabolites). Comparative genomics should be expanded to include additional strains from diverse tea waste sources (green tea, black tea, pu-erh tea) and geographic locations to assess functional gene conservation and biogeography. Synthetic community experiments with defined strain combinations would elucidate ecological interactions (synergy, competition, cross-feeding) and optimize consortium composition for maximal biomass conversion efficiency. Integration of genomic data with metabolomic and proteomic profiling would provide a systems-level understanding of tea waste degradation pathways and identify bottlenecks for process optimization [3,4,5].

### 4.7. Broader Implications for Waste Valorization and Circular Bioeconomy

The genomic characterization of tea waste-degrading bacteria contributes to the broader goal of developing sustainable bioprocesses for agricultural and food industry waste streams. Tea waste shares structural and chemical features with other lignocellulosic residues (coffee grounds, fruit pomace, brewery spent grains), and the strains and enzymes identified here may have applications beyond tea-specific substrates. The open CAZyme pangenome structure observed in this small strain collection suggests that systematic bioprospecting of composting environments could yield novel enzymes with improved thermostability, pH tolerance, or substrate specificity for industrial applications [1,2].

The low AMR burden and chromosomal localization of resistance genes in these environmental isolates contrast with the high prevalence of mobile ARGs in agricultural soils amended with manure or wastewater sludge. This distinction underscores the relevance of waste origin and treatment procedures to AMR risk. Composting of plant-derived waste streams may represent a lower-risk application for microbial inoculants compared to animal waste or clinical waste processing, although rigorous biosafety assessment remains essential [43].

The integration of genomic data with life cycle assessment (LCA) and techno-economic analysis (TEA) would support evidence-based decision-making for tea waste valorization strategies. Genomic information on enzyme profiles, growth requirements, and biosafety considerations informs bioprocess design parameters (inoculum composition, substrate pretreatment, fermentation conditions), which in turn determine capital costs, operating expenses, and environmental impacts. Multi-strain consortia designed using genomic data may achieve higher biomass conversion efficiency and product yields than single-strain inoculants, improving economic viability and environmental sustainability of tea waste valorization [4,11].

### 4.8. Mobilome and Horizontal Gene Transfer Potential in Composting Environments

The 349 MGE-associated genes identified across six tea waste isolates represent a substantial mobilome for a small, closed collection of environmental strains. The ubiquitous presence of IS3-family elements in all six genomes is consistent with the known global distribution of this IS family across diverse bacterial phyla and its documented role in genome rearrangements, gene inactivation, and promoter capture in environmental bacteria [35]. The strain-specific distribution of IS1595 and IS5/IS66 elements in *Pseudomonas* isolates, and IS256 elements in *Pseudomonas* and *Stenotrophomonas*, may reflect horizontal acquisition from shared environmental reservoirs during composting succession [34,35].

The detection of *traC* and/or *traG* conjugative transfer genes in three strains (*Stenotrophomonas*, *Pseudomonas*, and *Pantoea*) suggests the genetic potential for conjugative plasmid transfer that is not apparent from CARD-based AMR screening alone. Though conjugative transfer genes are identifiable, the fact that they are present does not imply active plasmid mobilization, and experimental confirmation of functional transfer is required via conjugation assays. The integron-associated *intA* recombinase in *Pantoea* sp. and class 1 integron structural genes in *Pseudomonas* isolates point to additional mechanisms for gene cassette acquisition and rearrangement [39]. Although the CARD-confirmed ARGs (*cat86*, *dfrG*, *oqxB*) showed no flanking MGEs within ±10 kb, the broader genome-wide mobilome demonstrates that the genomic context for potential future ARG acquisition exists in these strains. This is particularly notable for the *Stenotrophomonas* isolate, which harbors tet gene homologs, IS256-family elements, conjugative transfer genes (*traC*, *traG*), and a Tn-resolvase in a single genome. Conjugation assays and metagenomics-based monitoring of horizontal gene transfer dynamics across the fermentation time course are recommended to assess functional transfer potential before any open-environment deployment [18,35,43].

The Tn1721-type *tetR*–*tetA* module in *Stenotrophomonas* sp. is of particular note, as *Stenotrophomonas maltophilia* is recognized as an opportunistic pathogen and reservoir for horizontally transferable resistance elements in clinical settings. While the tea waste isolate showed minimal VFDB virulence factor content and ANI < 95% to *S. maltophilia* type strains, the co-occurrence of *tet* gene homologs, IS256-family elements, conjugative transfer genes (*traC*, *traG*), and a Tn-resolvase in a single genome warrants cautious evaluation before any open-environment deployment [18]. Conjugation assays and metagenomics-based monitoring of horizontal gene transfer dynamics across the fermentation time course are recommended to assess functional transfer potential [18,35,43].

## 5. Conclusions

This study reports high-quality draft genome sequences and comparative genomic analysis of six bacterial strains isolated from oolong tea waste fermentation, representing taxonomically diverse lineages with complementary lignocellulose-degrading capabilities. The high genome completeness (98.4–99.5%) and low contamination (0.5–1.2%), respectively, allow for good functional annotation and comparative studies, showcasing the quality of our assembly.

Three main findings emerge from this genomic characterization. First, the carbohydrate-active enzyme repertoire comprises 274 unique CAZyme families with an open pangenome structure, suggesting substantial functional diversity in plant biomass-degrading capacity. All strains harbor conserved core enzymes for cellulose and hemicellulose degradation, while accessory and unique CAZyme families reflect strain-specific adaptations. Descriptive comparisons point to potentially different enzyme profiles in relation to isolation time; however, these patterns must be further validated with experimental techniques (i.e., enzyme assays and substrate degradation) before making any conclusions about functional specialization.

Second, antimicrobial resistance gene screening using CARD, ResFinder, and AMRFinderPlus identified three chromosomally encoded ARGs (cat86, dfrG, oqxB) with no plasmid-associated determinants confirmed by these databases. Four strains encoded tetracycline resistance gene homologs (76.2–79.2% identity) below CARD detection thresholds, while conjugative transfer genes were detected in three of such strains. Conclusions about biosafety or strain deployment cannot be made until phenotypic antimicrobial susceptibility testing, conjugation assays, and metagenomics-based monitoring of horizontal gene transfer.

Third, the presence of virulence factor database annotations in isolates is mostly due to environmental adaptation (motility, biofilm formation, and iron acquisition) rather than established pathogenicity determinants. Database annotations alone cannot distinguish environmental fitness traits from clinically relevant virulence determinants, and experimental safety assessments are required.

This genomic dataset provides a reference for understanding bacterial adaptation to tea waste environments and establishes a foundation for rational strain selection in future experimental studies. However, the conclusion that these isolates are suitable candidates for controlled fermentation applications should be substantially qualified in the absence of functional enzyme assays, antimicrobial susceptibility testing, and experimental safety assessments. The combination of genomic data with physiological characterization, community ecology experiments, and bioprocess optimization will enable the development of efficient, sustainable, and safe microbial technologies for tea waste valorization in support of future circular bioeconomy applications.

## Figures and Tables

**Table 1:** Genome assembly statistics and taxonomic assignment of six bacterial strains isolated from oolong tea waste fermentation. Genome size, GC content, N50, predicted protein-coding genes, CheckM completeness and contamination estimates, average nucleotide identity (ANI) to closest type strain, and NCBI BioProject accession are shown. ANI ≥ 95% indicates a species-level match; ANI < 95% suggests potential taxonomic novelty. All assemblies met quality thresholds (completeness ≥ 95%, contamination ≤ 5%) for comparative genomic analysis.

Strain	Isolation Stage	Genome Size (Mbp)	GC Content (%)	N50 (kb)	Predicted Proteins	Completeness (%)	Contamination (%)	ANI (%)	Closest Reference	NCBI BioProject
***Bacillus* sp.**	Day 0	4.21	43.2	520	4123	99.1	0.8	92.3	*B. subtilis*	PRJNA1335891
***Paenibacillus* sp.**	Day 3	5.38	44.7	489	5201	98.4	1.2	98.2	*P. xylanexedens*	PRJNA1335891
***Pantoea* sp.**	Day 3	5.12	53.1	445	4876	99.2	0.9	96.4	*P. agglomerans*	PRJNA1335891
***Pseudomonas* sp. 1**	Day 28	6.15	61.8	578	6215	99.5	0.5	97.6	*P. putida*	PRJNA1335891
***Pseudomonas* sp. 2**	Day 35	6.33	61.4	561	6576	99.3	0.7	97.4	*P. putida*	PRJNA1335891
***Stenotrophomonas* sp.**	Day 35	3.61	66.5	600	3751	98.7	1.1	91.8	*S. maltophilia*	PRJNA1335891

**Table 2:** Antimicrobial resistance genes (ARGs) identified in bacterial isolates from tea waste fermentation. Gene name, antibiotic class, resistance mechanism, genomic location (chromosome or plasmid), and mobile genetic element (MGE) associations are shown. All three ARGs were chromosomally encoded with no flanking MGEs detected within ±10 kb. Identity and coverage values met CARD detection thresholds (≥80% identity, ≥80% coverage). Four strains (*Paenibacillus* sp., *Pseudomonas* sp. 1, *Pseudomonas* sp. 2, *Stenotrophomonas* sp.) contained no ARGs meeting detection criteria.

Strain	ARG Gene	Antibiotic Class	Resistance Mechanism	Genomic Location	Mobile Genetic Element
***Bacillus* sp.**	*cat86*	Chloramphenicol	Enzyme inactivation	Chromosome	None
***Bacillus* sp.**	*dfrG*	Trimethoprim	Target alteration	Chromosome	None
***Pantoea* sp.**	*oqxB*	Fluoroquinolone	Efflux pump	Chromosome	None

**Table 3:** Virulence factor (VF) annotation summary by functional category. Number of virulence-associated genes identified in each strain, categorized by function: motility (flagella, chemotaxis), biofilm formation (exopolysaccharide synthesis, adhesins), type III secretion system (T3SS) structural components, adhesion factors, siderophore biosynthesis, iron acquisition systems, toxins, and other regulatory/metabolic factors. VFs were identified using VFDB with BLASTP (E-value ≤ 1 × 10^−5^, identity ≥ 40%, coverage ≥ 50%). Many factors represent pleiotropic environmental adaptation traits rather than pathogenicity determinants.

Strain	Motility	Biofilm	T3SS	Adhesion	Siderophore	Iron Acq.	Toxin	Other	Total VFs
***Bacillus* sp.**	0	0	0	0	0	0	0	0	0
***Paenibacillus* sp.**	0	0	0	0	0	0	0	0	0
***Pantoea* sp.**	0	0	0	0	0	0	0	0	0
***Pseudomonas* sp. 1**	6	7	5	4	4	3	2	1	32
***Pseudomonas* sp. 2**	6	7	5	4	4	3	2	1	32
***Stenotrophomonas* sp.**	1	1	0	0	0	0	0	0	2

**Table 4:** Per-strain mobile genetic element (MGE) summary from genome-wide GFF3 annotation profiling. Number of CDS features assigned to each MGE category: IS elements/transposases, integron-associated genes, Tn-resolvase genes, and plasmid-associated transfer/replication genes (conjugal transfer proteins, mob, rep, relaxase, primase, helicase). Total MGE gene count per strain is shown. MGE assignment was performed by keyword-based parsing of Prokka GFF3 product annotations in R v4.5.2 with no prior biological assumptions; all six genomes contributed to a cumulative total of 349 MGE-associated coding sequences.

Strain	IS/Transposase Genes	Integron-Associated Genes	Tn-Resolvase Genes	Plasmid-Associated Genes	Total MGE Genes
***Bacillus* sp.**	8	5	3	37	53
***Paenibacillus* sp.**	12	5	0	32	49
***Pantoea* sp.**	15	6	0	30	51
***Pseudomonas* sp. 1**	21	19	0	30	70
***Pseudomonas* sp. 2**	33	22	0	32	87
***Stenotrophomonas* sp.**	12	4	1	22	39

## Data Availability

Raw sequencing data were deposited in the NCBI SRA (Sequence Read Archive) under BioProject accession PRJNA1335891 with the following reviewer link: https://dataview.ncbi.nlm.nih.gov/object/PRJNA1335891?reviewer=htdim7817ab5mla45h5baf5sg7 (accessed on 30 August 2026).

## Supplementary Materials

The following supporting information can be downloaded at: https://www.mdpi.com/article/10.3390/foods15183201/s1. Supplementary Figure S1: Schematic diagram of the reactor setup; Supplementary Figure S2: Photograph of the experimental setup; Supplementary Figure S3: Phylogenomic tree constructed using GTDB-Tk with relevant type strains from the Genome Taxonomy Database; Supplementary Figure S4: Genomic context analysis for identified antimicrobial resistance genes (ARGs) showing flanking regions (±10 kb); Supplementary Table S1: Complete genome assembly statistics for all six strains; Supplementary Table S2: Substrate-specific CAZyme gene counts for each strain; Supplementary Table S3: Genomic context analysis for identified antimicrobial resistance genes (ARGs); Supplementary Table S4: Cumulative CAZyme family accumulation across strains; Supplementary Table S5: Full GFF3-derived MGE feature table; Supplementary Table S6: IS element family assignments by strain; Supplementary Table S7: Per-strain genomic feature summary.
